# Characteristics of Intestinal Microbiota and Host Gene Regulation in *Coilia nasus* Responding to Stress

**DOI:** 10.3390/antiox14060626

**Published:** 2025-05-23

**Authors:** Jun Gao, Haojun Zhu, Jiancao Gao, Gangchun Xu

**Affiliations:** Freshwater Fisheries Research Center, Chinese Academy of Fishery Sciences, Wuxi 214081, China; gaojun@ffrc.cn (J.G.); zhuhaojun@ffrc.cn (H.Z.); gaojiancao@ffrc.cn (J.G.)

**Keywords:** stress response, intestinal microbiota, oxidative stress, ferroptosis, *Coilia nasus*

## Abstract

Transport stress in aquaculture poses significant challenges to fish health by inducing oxidative stress and intestinal damage. This study investigated the effects of transport stress on intestinal microbiota, host gene regulation, and metabolic responses in *Coilia nasus*. The fish were subjected to simulated transport conditions, followed by an analysis of their intestinal antioxidant capacity, inflammatory factors, transcriptome sequencing, metagenomic profiling, and metabolomic assays. The results revealed that transport stress significantly suppressed antioxidant enzyme activities (e.g., catalase, superoxide dismutase, glutathione peroxidase) and elevated oxidative damage (malondialdehyde, lipid peroxidation) alongside upregulating pro-inflammatory cytokines. The transcriptomic analysis identified differentially expressed genes enriched in the lipid metabolism and ferroptosis pathways, with the increased lipid peroxidation and iron overload activating ferroptosis. The metagenomic data showed an altered gut microbiota composition, including increased *Aeromonas* and reduced beneficial metabolites (e.g., propionic acid, bile acids). Correlation analyses linked the microbial shifts and metabolite changes to ferroptosis and barrier dysfunction. These findings demonstrate that transport stress disrupts intestinal redox balance, induces ferroptosis, and reshapes gut microbiota, collectively compromising intestinal integrity and health in *C. nasus*.

## 1. Introduction

Stress responses are physiological reactions triggered by various environmental factors. Excessive stress, however, can disrupt the balance between oxidative and antioxidative processes, thereby inducing oxidative stress [1]. The intestine is a vital digestive and immune organ of the body. When the body experiences external stimuli leading to a stress response, the intestine is often the first and most susceptible organ to be affected. This can result in damage to the intestinal barrier and disruption of immune function, and can subsequently trigger irritable bowel syndrome [2,3]. Oxidative stress disrupts the homeostasis of the intestinal environment, impairing the barrier function of the intestinal mucosa. This leads to dysfunction in the gut microbiota and increased permeability of the intestinal mucosa, and facilitates the adhesion and penetration of pathogens through the mucus layer into the intestinal tissues. The balance among gut microbial communities is disrupted, resulting in the overgrowth of Gram-negative bacteria and enhanced adhesiveness and invasive capacity toward the mucosa. Consequently, substantial amounts of endotoxins and other toxic substances are produced. These harmful substances enter the body, exacerbating further damage to the intestinal mucosal barrier while triggering immune and inflammatory responses [3,4]. It can be seen that the damage to intestinal barrier function and the gut microbiota disorders induced by oxidative stress are important factors leading to bodily injury and death.

Reactive oxygen species (ROS) generated by oxidative stress can directly damage intestinal epithelial cells, causing lipid peroxidation, protein oxidation, and DNA damage. This results in impaired cellular function and increased cell apoptosis, thereby compromising the integrity of the intestinal microbiota [5]. Oxidative stress activates pro-inflammatory signaling pathways such as nuclear factor kappa-B (NF-κB), leading to the production of pro-inflammatory cytokines. This inflammatory microenvironment further damages the intestinal tissue and impairs its function [6,7,8]. Furthermore, oxidative stress can disrupt the tight junctions between intestinal cells, thereby impairing the intestinal barrier and increasing gut permeability. Exposure to perfluoroalkyl and polyfluoroalkyl substances (PFAS) induces oxidative stress, which causes the breakdown of the mucous layer on the intestinal epithelium in crucian carp (*Carassius auratus*). This results in significant increases in pro-inflammatory cytokines and apoptosis-related genes [9]. Oxidative stress induced by various factors causes intestinal damage and inflammation in mirror carp (*Cyprinus carpio*) [10], *Girella laevifrons* [11], zebrafish (*Danio rerio*) [12], and *Lateolabrax maculatus* [13].

Oxidative stress alters the composition of the gut microbiota, reducing the abundance of beneficial bacteria and increasing the abundance of harmful bacteria, thereby exacerbating intestinal inflammation and damage [14]. Pathogenic bacteria can disrupt the tight junctions between cells, increase the permeability of intestinal epithelial cells, reduce the ability to defend against pathogens, and elevate the expression levels of pro-inflammatory cytokines such as interleukins and tumor necrosis factor, thereby inducing intestinal inflammatory responses [15]. Chronic inflammation and elevated oxidative stress in the intestine are closely associated with gut microbiota dysbiosis and metabolic disorders [16]. The imbalance of the gut microbiota reduces the production of short-chain fatty acids (SCFAs) and other beneficial metabolites [17]. SCFAs play a crucial role in maintaining intestinal health; their reduction weakens the barrier function of the intestine and impairs immune regulation [17]. PFAS-induced oxidative stress causes changes in the composition of the gut microbiota in carp, increasing the abundance of potential pathogens [9]. Exposure to microplastics (MPs) induces oxidative stress, leading to disruptions in the gut microbiota and metabolic disorders in zebrafish [12]. Similar findings have been observed in studies on common carp [18], striped catfish (*Pangasianodon hypophthalmus*) [19], freshwater grouper (*Acrossocheilus fasciatus*) [20], and rainbow trout (*Oncorhynchus mykiss*) [21].

*Coilia nasus* is a rare species of *Coilia* known for its strong stress response, which usually leads to high mortality. Previous studies on *C. nasus* have demonstrated that oxidative stress, apoptosis, and elevated mortality rates are induced by transport stress [22]. However, previous investigations have primarily focused on the liver and brain tissues and have not addressed the underlying regulatory mechanisms between the intestine and its microbiota [23,24]. Therefore, in our present study, we conducted RNA-seq, metagenomics, and metabolomics, and integrated them to elucidate the intestinal microbiota and host gene regulation in *C. nasus* responding to stress. Our findings will propose novel insights to the stress response in fish and promote healthier aquaculture practices.

## 2. Materials and Methods

### 2.1. Experimental Fish and Stress Experiment

The fish were sampled from temporary concrete ponds (7 m × 5 m × 1 m) using a seine net. They were pooled and randomly redistributed into six culture tanks (75 m × 55 m × 33 m). These fish were then randomly divided into two groups, with three replicates per group, each containing 30 healthy, undamaged individuals. The control group (C), consisting of three tanks, was not subjected to transportation stress. The remaining tanks were agitated for 10 s every 5 min to simulate transportation conditions. After an 8 h simulated transportation period, 20 fish from each tank were anesthetized using 70 mg/L MS-222. Following anesthesia, the fish were individually weighed (8.86 g ± 1.76 g) and measured for body length (136.98 mm ± 9.26 mm), and their intestinal tissues and contents were rapidly separated and stored in liquid nitrogen for molecular biological analysis at −80 °C upon return to the laboratory.

### 2.2. Intestinal Transcriptome Sequencing

For the method of metagenomic analysis, refer to [24]. RNA was extracted from the intestinal tissues (three samples each group) and qualified for library preparation following quality assessment. The library construction, including cDNA synthesis, end repair, A-tailing, and adapter ligation, was performed according to the Illumina protocol. cDNA fragments of 200–300 bp were size-selected and subjected to PCR amplification. The sequencing was conducted on the Illumina NovaSeq 6000 platform (Illumina, San Diego, CA, USA) (paired-end 150 bp). The raw data processing involved trimming and quality control using Trimmomatic (v0.32) with the parameters SLIDINGWINDOW:4:15 and MINLEN:75. The clean reads were mapped to the *C. nasus* reference genome (GCA_007927625.1) in orientation mode using hisat2, and the data quality was evaluated with qualimap_v2.2.1. The expression levels were quantified using the fragments per kilobase of exon model per million mapped fragments, and the differentially expressed genes (DEGs) were identified with edgeR in R, applying the criteria of |log2 fold change| >1 and false discovery rate <0.05. Finally, the Kyoto Encyclopedia of Genes and Genomes (KEGG) pathway analysis was performed using KOBAS, with significance determined by Bonferroni-corrected *p*-values < 0.05.

### 2.3. Metagenomic Sequencing of Intestinal Microbiota

For the method of metagenomic analysis, refer to [25,26]. In brief, microbial DNA was extracted. Shotgun metagenomic libraries (five libraries for each group) were prepared and sequenced on the Illumina NovaSeq 6000 platform (PE150 mode) at Biozeron Biological Technology Co., Ltd. (Shanghai, China). The raw reads were quality-trimmed using Trimmomatic v0.36 to remove adapters and low-quality sequences. The taxonomic classification of the clean reads was performed with Kraken2 using a custom database (NCBI RefSeq, release 20221209) at seven taxonomic levels. Bracken v2.7.0 was used to estimate species- and genus-level abundances. The clean reads were assembled into contigs (≥500 bp) using MegaHit (-min-contig-len 500), and open reading frames (ORFs) were predicted with METAProdigal. The ORFs were clustered using CD-HIT to generate a unique gene set, with the longest sequence representing each gene. Gene abundance was calculated in transcripts per million by aligning the reads to the unique gene set (BWA-MEM) with ≥50 bp alignment length and ≥95% identity. Functional annotations were performed by searching against KEGG (kofam v1.2.0), the Carbohydrate-Active enZYmes Database, eggNOG, the Pathogen Host Interactions Database (BLASTP, e-value ≤10^−5^, ≥70% identity), and Virulence Factors of Pathogenic Bacteria (BLASTP, e-value ≤10^−5^, ≥70% identity).

### 2.4. Metabolites of Intestinal Microbiota

For the method of metabolomic analysis, refer to [27]. In brief, metabolite extraction (six samples from each group) was performed. LC/MS analysis was conducted using a Waters UPLC system (waters, Shanghai, China) coupled with a Xevo G2-XS QTOF mass spectrometer (waters, Shanghai, China) in both positive and negative ion modes. Data acquisition in MSe mode allowed simultaneous low- and high-collision energy collection. The raw data were preprocessed with Progenesis QI and annotated using METLIN and an internal database. The theoretical fragmentation and mass deviation thresholds were set at <100 ppm. The data were normalized, and the reproducibility was assessed via PCA and Spearman correlation. The metabolites were classified using KEGG and LipidMaps databases. Statistical significance was determined by the *t*-test, and orthogonal partial least squares discriminant analysis (OPLS-DA) modeling was applied to identify the differential metabolites (FoldChange [FC] > 1, *p* < 0.05, Variable Importance in the Projection [VIP] > 1).

### 2.5. Detection of Intestinal Physiological Indexes

The activity of total antioxidant capacity (T-AOC), catalase (CAT), superoxide dismutase (SOD), and glutathione peroxidase (GSH-Px), as well as the concentrations of free fatty acid (FFA), ferric, lipid peroxidation (LPO), and malondialdehyde (MDA), was determined using commercial test kits (Nanjing Jiancheng Bioengineering Institute, Nanjing, China), following the manufacturer’s instructions.

### 2.6. Real-Time Fluorescence Quantitative PCR for Gene Expression Analysis

To verify the RNA-Seq results, 10 DEGs were assessed by RT-qPCR. To verify the variation trends of the DEGs detected by Illumina, the RT-qPCR data were given as a log_2_ fold change. Additionally, the mRNA expression of acyl-CoA synthetase long chain family member 4 (*acsl4*) and glutathione peroxidase 4 (*gpx4*) was also detected, which was analyzed in triplicate, and the expression of the target genes was calculated using the 2^−△△CT^ method. The primers used for the RT-qPCR are shown in Table S1 (see Supplementary Materials). *β-actin* was used for the housekeeper gene.

### 2.7. Statistical Analysis

All experimental data were subjected to Student’s *t*-test using SPSS version 26.0 (IBM Corp., Armonk, NY, USA). The results are presented as the mean ± standard deviation (SD). Statistical significance was determined at a significance level of *p* < 0.05.

## 3. Results

### 3.1. Effect of Transport Stress on Intestinal Inflammation and Antioxidant Indexes in Coilia nasus

**Figure 1:**
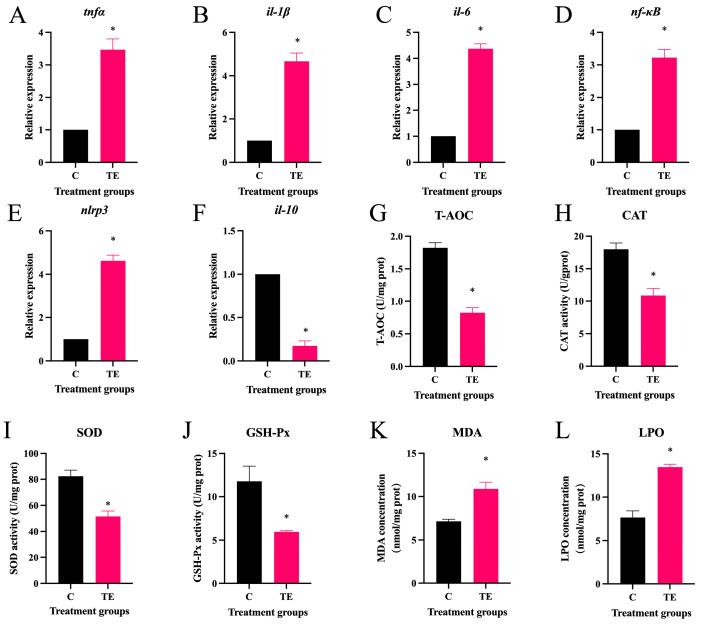
Effect of transport stress on intestinal inflammation and antioxidant indexes in *Coilia nasus*. Intestinal inflammation indexes: relative expression of *tnfα* (**A**), *il-1β* (**B**), *il-6* (**C**), *nf-κB* (**D**), *nlrp3* (**E**), and *il-10* (**F**); Antioxidant indexes: activity of T-AOC (**G**), CAT (**H**), SOD (**I**), and GSH-Px (**J**); concentration of MDA (**K**) and LPO (**L**). The results are shown as means ± SE. *n* = 9 per group. * indicates significant difference.

After transport stress, the mRNA expression of the pro-inflammatory factors tumor necrosis factor α (*tnfα*) (Figure 1A), interleukin (*il-1β*) (Figure 1B), *il-6* (Figure 1C), *nf-κb* (Figure 1D), and NLR family pyrin domain containing 3 (*nlrp3*) (Figure 1E) in the intestine of *C. nasus* significantly increased (*p* < 0.05), while the mRNA expression of anti-inflammatory factor *il-10* significantly decreased (*p* < 0.05) (Figure 1F). In addition, after transport stress, the activities of the oxidative-stress-related indicators T-AOC (Figure 1G), CAT (Figure 1H), SOD (Figure 1I), and GSH-Px (Figure 1J) in the intestine of *C. nasus* significantly decreased (*p* < 0.05), while the level-of-damage indicators MDA (Figure 1K) and LPO (Figure 1L) significantly increased (*p* < 0.05).

### 3.2. Transcriptomic Analysis of Intestine in C. nasus Under Transport Stress

The response of the intestinal genes to transport stress was analyzed via transcriptome sequencing. A total of 2280 differentially expressed genes (DEGs) were identified, including 1012 upregulated DEGs and 1268 downregulated DEGs. As shown in Figure S1 (see Supplementary Materials), the expression patterns of the RT-qPCR and RNA-Seq displayed general agreement. The tendency between the two methods was consistent.

**Figure 2:**
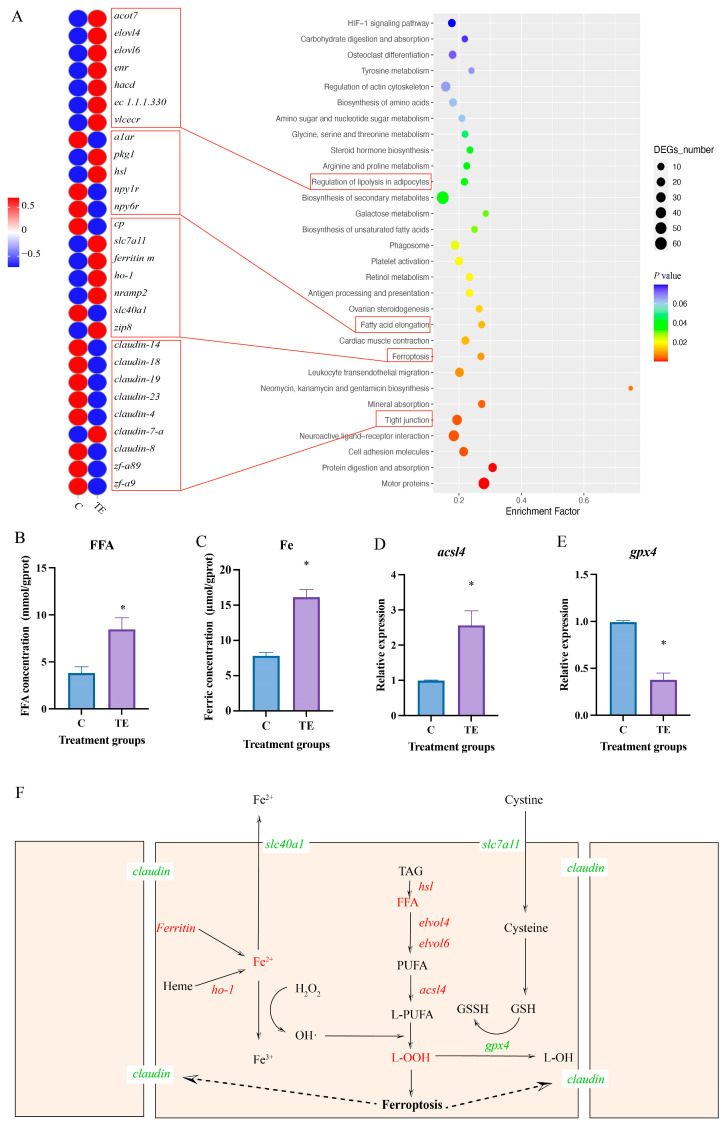
Characteristics of intestinal gene regulation in *C. nasus* responding to transport stress. KEGG pathway enrichment and heat map of differentially expressed genes in *C. nasus* under transport stress (**A**). Ferroptosis-related indexes: concentration of FFA (**B**) and ferric (**C**); relative expression of *acsl4* (**D**) and *gpx4* (**E**). The results are shown as means ± SE. *n* = 9 per group. * indicates significant difference. Schematic diagram of the regulatory mechanism linking lipid metabolism, ferroptosis, and tight junction (**F**); red words indicate upregulation, green words indicate downregulation. Solid lines represent regulatory relationships and dashed lines represent potential effects.

Through KEGG enrichment analysis, these DEGs were enriched in the pathways of regulation of lipolysis in adipocytes, fatty acid elongation, ferroptosis, and tight junction (Figure 2A). Moreover, the DEGs enriched in regulation of lipolysis in adipocytes, fatty acid elongation, and ferroptosis were upregulated, while those enriched in fight junction were downregulated (Figure 2A). Lipid metabolism is closely related to ferroptosis. After transport stress, the concentrations of FFA (Figure 2B) and ferric ion (Figure 2C) in the intestines of *C. nasus* significantly increased (*p* < 0.05). The mRNA expression of the key ferroptosis indicator *acsl4* significantly increased (*p* < 0.05) (Figure 2D), while the mRNA expression of *gpx4* significantly decreased (*p* < 0.05) (Figure 2E). The association mechanisms among the above four pathways are shown in Figure 2F. The oxidative stress induced by transport stress led to lipid metabolism disorders and ferric ion overload, which activated ferroptosis in the intestine of *C. nasus* and subsequently inhibited the tight junctions between intestinal cells.

### 3.3. Changes of Intestinal Microbial Communities in C. nasus Under Transport Stress

**Figure 3:**
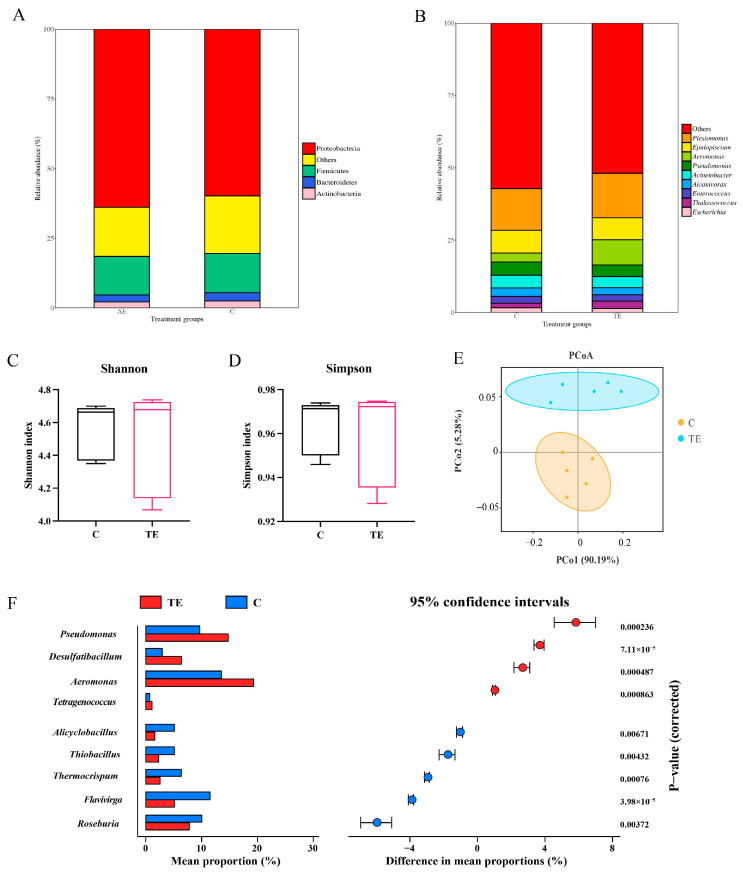
Changes of intestinal microbial communities in *C. nasus* under transport stress. Composition profiles of intestinal microbiota at phylum level (**A**) and genus level (**B**). Intestinal microbiota diversity: Shannon (**C**) and Simpson (**D**) indexes; principal co-ordinates analysis (**E**). Differences in intestinal microbiota based on statistical analysis of the metagenomic profiles analysis (**F**).

In the C group and the TE group, the relative abundances of Proteobacteria and Firmicutes were higher, with the relative abundance of Proteobacteria in the TE group (63.2%) being higher than that in the C group (59.7%) (Figure 3A). In the C group and the TE group, the relative abundances of *Plesiomonas* and *Epulopiscium* were higher. The relative abundance of *Aeromonas* in the TE group (8.7%) was higher than that in the C group (3.1%) (Figure 3B). There was no significant difference in the Shannon and Simpson indices between the C group and the TE group (Figure 3C,D). In the principal co-ordinates analysis (PCoA), the distance between the two groups of samples was relatively far, indicating that there was a large difference in the microbial community structure of the two groups (Figure 3E). Based on the stamp analysis, the relative abundances of *Pseudomonas*, *Desulfatibacillum*, *Aeromonas*, and *Tetragenococcus* in the TE group were significantly higher than those in the C group (Figure 3F). The relative abundances of *Flavivirga*, *Alicyclobacillus*, *Thiobacillus*, *Thermocrispum*, and *Roseburia* in the TE group were significantly lower than those in the C group (Figure 3F).

### 3.4. Metabolic Alterations of Intestinal Microbiota in C. nasus Under Transport Stress

**Figure 4:**
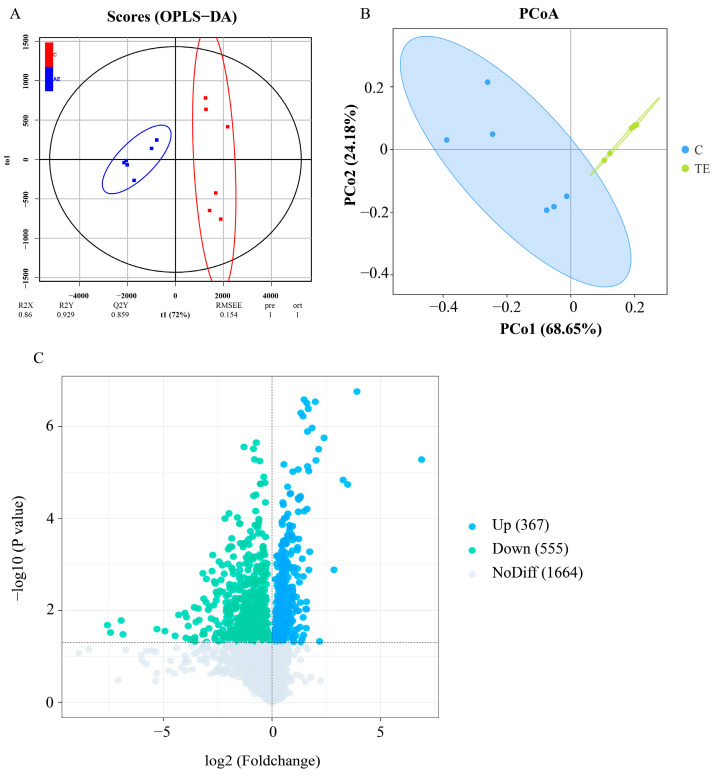
Metabolic alterations of intestinal microbiota in *C. nasus* under transport stress. OPLS-DA score plot between the C and TE groups (**A**). PCA analysis between the C and TE groups (**B**). Volcano plot of differential metabolites between the C and TE groups (**C**).

The metabolome analysis was used to characterize the changes in the intestinal microbiota metabolites after transport stress. The OPLS-DA score plot showed a clear separation of metabolic features between the C group and the TE group (Figure 4A). Principal component analysis (PCA) revealed a large distance between the C group and the TE group samples, indicating significant differences in the metabolites between the two groups (Figure 4B). A total of 2586 metabolites were identified, among which 367 metabolites were significantly upregulated and 555 metabolites were significantly downregulated (Figure 4C). In addition, based on reports from other studies, we screened out six differential metabolites that are beneficial to intestinal health (Table 1). Compared with the C group, the abundance of tryptophan metabolic derivatives (3-indoleacetic acid, indole-5-acetic acid, indole-1-propanoic acid, indole-3-carboxylic acid), bile acids, and propionic acid in the TE group was significantly downregulated (Table 1).

### 3.5. Correlation Analysis

**Figure 5:**
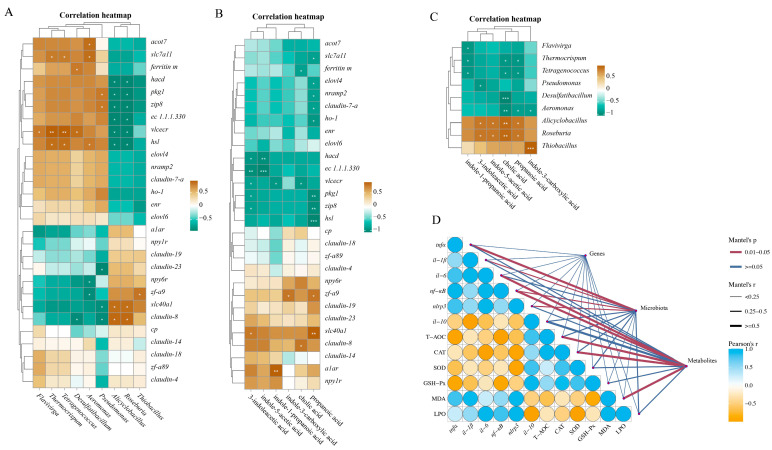
Correlation analysis. Correlation analysis between differentially expressed genes and differences microbiota based on Pearson (**A**). Correlation analysis between differentially expressed genes and differences metabolites based on Pearson (**B**). Correlation analysis between differences microbiota and differences metabolites based on Pearson (**C**). Mental test analysis between inflammatory and antioxidant indicators, differentially expressed genes, differential microbiota, and differential metabolites (**D**). The difference was considered statistically significant when * *p* < 0.05, ** *p* < 0.01, or *** *p* < 0.001.

The correlations between inflammation, antioxidant indicators, DEGs, difference microbiota, and difference metabolites were analyzed using the Pearson and Mantel tests. There was a correlation between the difference microbiota and the DEGs. *Roseburia* showed a significant negative correlation with lipid-metabolism-related genes (*hsl*, *vlcecr*, *hadc*, *pkg1*) (Figure 5A). *Pseudomonas* and *Aeromonas* showed a significant positive correlation with lipid-metabolism-related genes (*acot7*, *hsl*, *pkg1*) and ferroptosis-related genes (*slc7a11*, *zip8*) (Figure 5A). There was a correlation between the difference metabolites and the DEGs. Propionic acid showed a significant positive correlation with lipid-metabolism-related genes (*elovl4*, *hsl*, *pkg1*) and ferroptosis-related genes (*slc7a11*, *nramp2*, *zip8*, *ho-1*) (Figure 5B). As shown in Figure 5C, there was a correlation between the difference metabolites and the difference microbiota. The Mantel test analysis showed that there was no significant correlation between the DEGs and inflammation, which are both antioxidant indicators (Figure 5D). The difference microbiota was significantly correlated with inflammation, while the difference metabolites were significantly correlated with both inflammation and antioxidant indicators (Figure 5D).

## 4. Discussion

Transport-associated physiological stress remains a critical challenge in intensive aquaculture practices, precipitating a pathophysiological cascade that compromises piscine immunity and survival [28]. Mechanistically, transit-induced activation of the hypothalamic–pituitary–interrenal (HPI) axis drives cortisol hypersecretion, which disrupts the mitochondrial redox balance via the inhibition of electron transport chain Complexes I and III, thereby amplifying ROS generation. Empirical evidence across species demonstrates this redox disequilibrium: largemouth bass (*Micropterus salmoides*) exhibited a 42% elevation in hepatic SOD activity post-12 h transport, yet MDA accumulation revealed systemic antioxidant insufficiency [29]. Similar effects are seen in other fish species like *Megalobrama amblycephala* and *Ictalurus punctatus*, with reduced antioxidant capacities post-transport [30,31]. In this study, transport stress significantly suppressed the activities of CAT, SOD, GSH-Px, and T-AOC in the intestines of *C. nasus*, while markedly elevating MDA and LPO levels, indicative of oxidative stress induction in the intestinal tissues. Furthermore, the interplay between oxidative stress and inflammatory responses was amplified. ROS directly activated inflammatory responses via the NF-κB and mitogen-activated protein kinase (MAPK) signaling pathways. In the NF-κB pathway, ROS facilitated the phosphorylation of IκB kinase, releasing NF-κB to translocate into the nucleus, thereby initiating the transcription of pro-inflammatory factors such as TNF-α and IL-1β. For instance, in transported rainbow trout, IL-8 expression in the muscle increased 3.2-fold, and TLR22 was upregulated 2.7-fold [32]. Mitochondrial ROS released mtDNA fragments that bound to NLRP3 to form inflammasomes, promoting caspase-1-mediated IL-1β maturation [33]. Our results demonstrated the significant upregulation of intestinal pro-inflammatory mediators (*tnfα*, *il-1β*, *il-6*, *nf-κb*, and *nlrp3*) in *C. nasus* subjected to transport stress, mechanistically confirming stress-induced enteric inflammation. This inflammatory priming aligns with conserved piscine stress adaptation strategies, as evidenced in rainbow trout, where transportation provoked IL-1β-, IL-8-, and TNF-α-dominated immunoregulatory responses, mirroring the cytokine-mediated environmental acclimatization pathways across teleost species [32].

In this study, the transcriptomic analysis of the intestine of *C. nasus* revealed that differentially expressed genes were primarily enriched in the lipid metabolism and ferroptosis signaling pathways. Ferroptosis, a form of non-apoptotic cell death driven by iron-dependent lipid peroxidation, has emerged as a focal point in fish biology and disease research in recent years. Its core mechanism involves abnormal iron metabolism, the depletion of glutathione (GSH), the inhibition of GPX4 activity, and the accumulation of peroxidized phospholipid products [33]. Iron metabolic imbalance is a key trigger of ferroptosis. Transport stress was found to upregulate the expression of *ferritin* and *ho-1* in the intestine of *C. nasus* while downregulating *slc40a1*. Ferritin and HO-1 exacerbate lipid peroxidation via Fe^2^⁺ [34,35]. SLC40A1 is the sole known cellular iron exporter that maintains iron homeostasis by exporting Fe^2^⁺ [36]. These results indicate that the excessive iron ion release and impaired export led to iron metabolic imbalance in the intestinal cells of *C. nasus*. The iron overload participated in Fenton reactions, generating ROS that drove lipid peroxidation [37]. The oxidative stress exacerbated the accumulation of lipid peroxides. The transcriptomic analysis revealed that transport stress upregulates the expression of lipid-metabolism-related genes (*hsl*, *elovl*, *acsl4*) in the intestine of *C. nasus*. During ferroptosis, HSL, ELOVL, and ACSL4 form a metabolic cascade. HSL breaks down triglycerides to release PUFA; ELOVL elongates PUFA into longer-chain forms; and ACSL4 activates PUFA and facilitates its integration into membrane phospholipids, ultimately triggering lipid peroxidation and cell death [38]. Moreover, transport stress was found to increase the levels of MDA and LPO in the intestinal cells of *C. nasus*, indicating that oxidative stress induced by transport stress led to the excessive accumulation of lipid peroxides. This, in turn, activated ferroptosis. The collapse of the antioxidant system is another crucial aspect of ferroptosis. In this study, transport stress significantly downregulated the expression of the *gpx4* gene and the levels of GSH-Px in the intestine of *C. nasus*. As GPX4 is a core regulatory protein of ferroptosis, the inhibition of GPX4 activity directly results in the accumulation of intracellular lipid peroxidation, thereby inducing ferroptosis [39]. GSH, a key antioxidant, directly scavenges ROS like superoxide anions, hydrogen peroxide, and hydroxyl radicals within cells. Additionally, GSH acts as a substrate for GPX, facilitating the reduction of lipid hydroperoxides (LOOH) to non-toxic lipid alcohols (LOH), thereby alleviating oxidative-stress-induced cellular damage. This dual role of GSH underscores its importance in maintaining cellular redox homeostasis and protecting against oxidative injury [40]. These results indicated that the transport-stress-induced oxidative stress led to GSH depletion and the inhibition of GPX4 activity, thereby activating ferroptosis in the intestinal cells of *C. nasus*. Additionally, the transcriptomic analysis revealed that the transport stress significantly downregulated the expression of tight-junction-related genes such as *claudin* in the intestine of *C. nasus*. Studies have shown that ferroptosis and tight junctions exhibit a complex bidirectional regulatory relationship, jointly participating in the maintenance of intestinal barrier function and disease progression. During ferroptosis, ROS and lipid peroxidation products (e.g., MDA) directly attack tight junction proteins (e.g., Claudin-1, Occludin, ZO-1), leading to their downregulated expression or structural abnormalities [41]. This damage to the tight junction structures allows pathogen-associated molecules (e.g., lipopolysaccharides [LPS], bacterial toxins, and pro-inflammatory factors [e.g., IL-6, TNF-α]) to leak into the intestinal tissue, further accelerating the progression of ferroptosis [42]. The transport stress disrupted intestinal lipid homeostasis in *C. nasus*, promoting lipid peroxide accumulation, ferroptosis activation, and tight junction compromise.

The gut microbiota of aquatic animals is predominantly constituted by Proteobacteria, Firmicutes, Bacteroidetes, and Actinobacteria [43]. Under normal conditions, these microbial communities maintain a dynamic equilibrium within the gut and contribute to various host physiological functions, including food digestion, nutrient absorption, and immune regulation. Transport stress disrupts this equilibrium, leading to reduced diversity and richness of the gut microbiota. In this study, while no significant differences in alpha diversity were observed between the groups, significant differences were detected in beta diversity. This suggests that transport stress did not alter the richness or evenness of the gut microbes but significantly affected the composition and distribution of the intestinal microbiota in *C. nasus*. These findings are consistent with previous studies on the effects of transport stress on the gut microbiota diversity and structure of largemouth bass [29]. Altered community structures can lead to abnormalities in host physiological functions [44]. The correlation analysis revealed significant associations between the differential microbes at the genus level and intestinal ferroptosis- and lipid-metabolism-related genes. However, the relationship between them required further research to confirm. Gut microbiota perform various functions within the host’s gut and are closely associated with host health [44,45]. After transport stress, the relative abundance of *Pseudomonas* and *Aeromonas* in the intestine of *C. nasus* significantly increased. Lipopolysaccharide (LPS), an important component of the cell wall of *Pseudomonas aeruginosa*, can trigger inflammatory responses and tissue damage and assist bacteria in evading the host’s immune defenses [45]. In aquaculture, *Aeromonas* is one of the most common pathogenic bacteria, causing various diseases in fish, such as bacterial septicemia [46]. Reconstruction of gut microbiota community structures alters microbial metabolite abundance, thereby modulating host intestinal gene expression and potentially affecting physiological functions [23]. The correlation analysis indicated that the increased relative abundances of *Pseudomonas* and *Aeromonas* might suppress the generation of microbial metabolites like propionic acid and bile acids. SCFAs can enhance the expression of tight junction proteins Claudin-1 and Occludin, thereby maintaining intestinal barrier integrity. Moreover, SCFAs modulate key antioxidant factors such as Kelchlike ECH-associated protein-1 (Keap1) and Nuclear Factor Erythroid 2-Related Factor 2 (Nrf2), protecting cells from ferroptosis-induced damage [47,48]. Transport stress significantly reduced the abundance of propionic acid in the gut microbiota of *C. nasus*. The downregulated SCFAs were significantly correlated with the expression of genes related to intestinal ferroptosis and lipid metabolism. These results suggested that transport stress reconstructed the community structure of gut microbiota and induced metabolic reprogramming, which might be associated with intestinal ferroptosis. However, the regulatory relationship between these processes requires further investigation.

While this study identified significant correlations between gut microbiota dysbiosis, metabolic alterations, and ferroptosis in *C. nasus* under transport stress, the mechanistic causality underlying these associations remains unresolved. For instance, although Pseudomonas and *Aeromonas* were linked to lipid-metabolism- and ferroptosis-related genes, the direct role of these bacteria in driving oxidative damage or regulating host pathways was not experimentally validated. Similarly, the observed reduction in beneficial metabolites could theoretically impair intestinal barrier function and exacerbate ferroptosis, but their specific molecular interactions with host antioxidant systems or tight junction proteins remain uncharacterized. Future studies should integrate microbial transplantation, metabolite supplementation, or targeted gene silencing to establish causal relationships and elucidate the precise microbial–host crosstalk mediating these processes.

## 5. Conclusions

Transport stress in *C. nasus* triggered oxidative stress, inflammation, and ferroptosis, leading to intestinal damage. The key findings included suppressed antioxidant enzyme activities (CAT, SOD, GSH-Px), elevated oxidative markers (MDA, LPO), and upregulated pro-inflammatory cytokines (*tnfα*, *il-1β*, *nf-κb*). Transcriptomic analysis revealed the enrichment of lipid metabolism and ferroptosis pathways, driven by iron overload and lipid peroxidation, alongside downregulated tight junction genes. The gut microbiota shifted toward pathogens (*Aeromonas*, *Pseudomonas*) with reduced beneficial metabolites (e.g., propionic acid, bile acids), correlating with intestinal barrier dysfunction. While correlations between microbiota dysbiosis, metabolic changes, and ferroptosis were identified, direct mechanistic links—such as the microbial regulation of host lipid metabolism or antioxidant pathways—remain unproven. Future studies should employ microbial transplantation, metabolite supplementation, or gene-editing approaches to validate causality and clarify molecular interactions. These findings highlight the need for strategies targeting oxidative balance, microbial modulation, and ferroptosis inhibition to mitigate transport-related stress in aquaculture.

## Figures and Tables

**Table 1:** Positive metabolic alterations in *C. nasus* under transport stress.

Name	log2FC	*p*-Value	VIP	Regulated
3-indoleacetic acid	−0.46297269	0.005879618	1.454613619	down
indole-5-acetic acid	−1.654871937	0.006137787	1.388295277	down
indole-1-propanoic acid	−0.658598224	0.037968101	1.084461372	down
indole-3-carboxylic acid	−0.178969357	0.030991001	1.086786359	down
cholic acid	−0.440879157	0.000378487	1.821650945	down
propanoic acid	−5.600468707	1.14 × 10^−10^	2.886101393	down

## Data Availability

The sequences were submitted to the NCBI SRA database (PRJNA1018811, PRJNA949809).

## Supplementary Materials

The following supporting information can be downloaded at: https://www.mdpi.com/article/10.3390/antiox14060626/s1, Figure S1: RNA-seq results verified via RT-qPCR; Table S1: primers used for RT-qPCR verification.

## Abbreviations

The following abbreviations are used in this manuscript:

acot7: Cytosolic acyl coenzyme A thioester hydrolase; elvol4: elongation of very long chain fatty acids protein 4; elvol6: elongation of very long chain fatty acids protein 6; enr: enoyl-[acyl-carrier-protein] reductase, mitochondrial; hacd: very-long-chain (3R)-3-hydroxyacyl-CoA dehydratase 1; ec 1.1.1.330: very-long-chain 3-oxoacyl-CoA reductase; vlcecr: very-long-chain enoyl-CoA reductase; a1ar: adenosine receptor A1; pkg1: cGMP-dependent protein kinase 1; hsl: hormone-sensitive lipase; npy1r: neuropeptide Y receptor type 1; npy6r: neuropeptide Y receptor type 6; zf-a89: claudin-like protein ZF-A89; zf-a9: claudin-like protein ZF-A9; cp: ceruloplasmin; slc7a11: cystine/glutamate transporter; ferritin m: ferritin, middle subunit; ho-1: heme oxygenase; nramp2: natural resistance-associated macrophage protein 2; slc40a1: solute carrier family 40 member 1; zip8: zinc transporter ZIP8.
