# Supplementary material for: Characteristics of Intestinal Microbiota and Host Gene Regulation in Coilia nasus Responding to Stress
Source: Antioxidants (Basel). 2025 May 23;14(6):626. doi: 10.3390/antiox14060626 (PMC12189889; doi:10.3390/antiox14060626)
Supplement: Supplementary file 1 [file antioxidants-14-00626-s001.zip › antioxidants-3631642-supplementary.pdf]

Table S1 primers used for RT-qPCR verification

Gene ID	Forward primer	Reverse primer	Tm °C (F)	Tm °C (R)	production length (bp)
β-actin	AACGGATCCGGTATGTGCAAAGC	GGGTCAGGATACCTCTCTTGCTCTG	60.05	59.65	124
elovl4	CCACCGACAACCGCCTGGAC	CCAGGAGTGCGGAGAGGGCT	59.97	59.97	180
hsl	GCCTGCGACACCCACCTGTC	CAGACGCCACGCACCCACAT	59.97	60.11	262
slc7a11	CGCAAGCGTGCACAGGAGGA	CCGGCTCCTGCACTGCCATC	59.98	60.11	241
ferritin	CCCCAACGCTGCTGCCTACC	GCTGGTGCTGGAGTGCCTGG	60.04	59.97	226
ho-1	CAGCAGGGCACGGCCAATCA	CCCGCTCTGGGAAGCACAGC	59.97	60.04	286
claudin-14	GTCTGCAGGGCGGCAGGAAG	TGAGCCCCTCGGGCCTGTTT	60.04	60.11	231
claudin-18	CCAGCGAACGAGCCCGACAG	TGCAGCGCACATACCAGGGC	60.11	59.98	315
claudin-19	CGCAAGCGTGCACAGGAGGA	CCGGCTCCTGCACTGCCATC	59.98	60.11	241
claudin-23	TGTCTGACGGGCGGGAGGAG	AACGAGCGCGTCACCACCTG	59.97	59.98	211
claudin-4	GTGGTCACGGTGGTGGCGAG	CGCCACCATGACACCCACCC	59.98	59.97	208

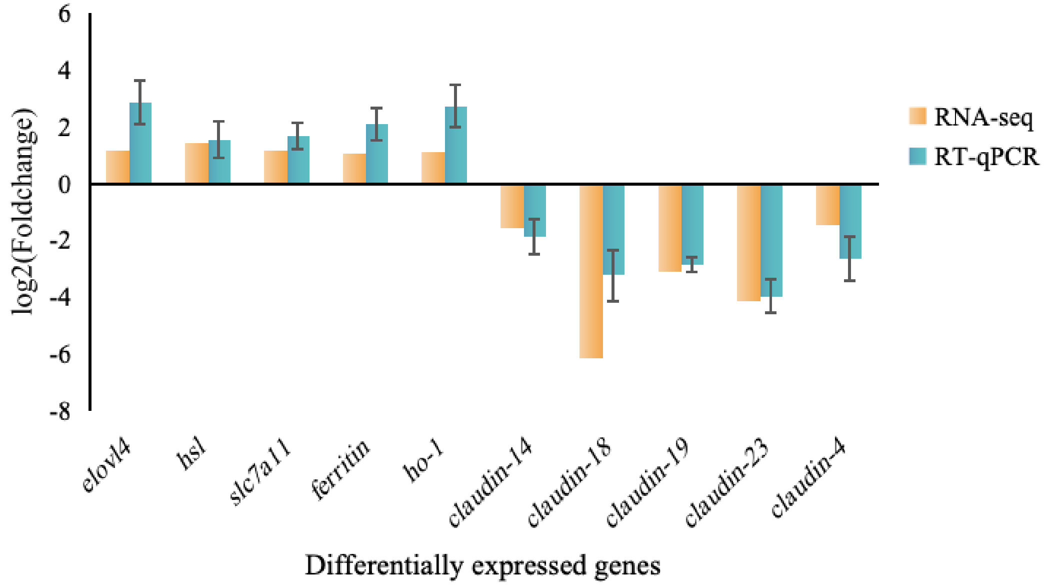

Figure S1. RNA-seq results verified via RT-qPCR.
